# Nutrition and Gut Health: Recent Advances and Implications for Development of Functional Foods

**DOI:** 10.3390/ijms241210075

**Published:** 2023-06-13

**Authors:** Guoyao Wu

**Affiliations:** Departments of Animal Science and Medical Physiology and Faculty of Nutrition, Texas A&M University, College Station, TX 77843, USA; g-wu@tamu.edu

## 1. Introduction

The small intestine is a highly differentiated and complex organ with many nutritional, physiological, and immunological functions [1,2,3,4,5,6,7,8]. First, the small intestine is responsible for the terminal digestion and absorption of dietary nutrients and is, therefore, essential to health, growth, development, reproduction, and sustaining life in organisms. Second, the neonatal small intestine can absorb immunoglobulins from milk before *gut closure*, which is important for the immunity of newborns. Third, the gut separates the internal milieu of organisms from their external environment, which is critical for the exclusion of food-borne pathogens and preventing the translocation of luminal microorganisms into the blood circulation. Finally, as the largest lymphoid organ in mammals, the small intestine participates in the immune surveillance of the intestinal epithelial layer and the regulation of mucosal responses to foreign antigens. In contrast to the small intestine, the large intestine harbors large amounts of microorganisms that ferment carbohydrates, proteins, and amino acids (AAs) to form short-chain fatty acids, H_2_S, ammonia, indoles, skatole, and other metabolites. In addition, unlike the small intestine, the large intestine is susceptible to the development of tumors. Thus, nutrients are major factors affecting gut health and function, as well as whole-body metabolism and homeostasis in humans and animals. Some of these advances are highlighted in this Special Issue of the *International Journal of Molecular Sciences* entitled “Nutrition and Gut Health” [1,2,3,4,5,6,7,8]. Its specific topics include the roles of dietary carbohydrates [1,2], AAs [3,4], lipids [5,6], and non-nutrient bioactive molecules [7,8] in animal models such as pigs, rats, and mice.

## 2. Effects of Dietary Carbohydrates on Intestinal Microbiota and Anti-Inflammatory Responses

In nutrition, polysaccharides are classified into starch, resistant starch, and nonstarch polysaccharides [2]. The latter can be either soluble or insoluble in water. Over the past three decades, interest has been growing in the role of oat bran (the hard outer layer of the oat grain) in intestinal health and preventing colon cancer. In this food, ~50% of the total dietary fiber is water-soluble (primarily *β*-glucan) and the other half is water-insoluble (mainly cellulose, lignin, and associated hemicelluloses). The consumption of β-glucans may play a role in reducing the intestinal absorption of dietary cholesterol and, therefore, preventing cardiovascular diseases. However, how oat bran influences nutrient digestibility, intestinal microbiota, and inflammatory responses in the hindgut of humans is largely unknown. Using the pig model (with a mean body weight of 30.5 kg), He et al. [1] found that, compared with the control group (fed a corn- and soybean-meal-based diet), dietary supplementation with 10% oat bran reduced the apparent total-tract digestibility of dietary gross energy (−1.9%), dry matter (−2.0%), organic matter (−1.8%), and crude protein (−3.1%) on day 14 of the study but had no effect on day 28 of the study. This suggests that pigs can adapt to the dietary intake of oat bran. Notably, dietary supplementation with oat bran for 28 days increased the abundances of *Prevotella* (3.2-fold), *Butyricicoccus* (2.3-fold), and *Catenibacterium* (12-fold), as well as the concentration of propionate (59%) in the colonic digesta, while reducing the abundances of *Coprococcus* (−42%) and *Desulfovibrio* (−72%) in the colonic digesta, compared with the control group. Thus, oat bran can profoundly modulate the composition of bacteria in the large intestine. Furthermore, dietary supplementation with oat bran for 28 days decreased the abundances of mRNAs for interleukin-8 (an inflammatory cytokine; −51%) in the cecal mucosa, as well as interleukin-8 (−30%), nuclear factor-κB (−46%), and tumor necrosis factor-α (−37%) in the colonic mucosa. Collectively, these results indicate that dietary supplementation with 10% oat bran for 4 weeks did not affect the apparent digestibility of dietary nutrients or energy but promoted the growth of cellulolytic bacteria for fiber fermentation and alleviated inflammatory responses in the mammalian hindgut.

Resistant starch (RS) is a polysaccharide that resists digestion in the small intestine and ferments in the large intestine [2]. This type of carbohydrate may reduce the risk of colorectal cancer in humans [9]. It was unknown whether RS from different sources may affect the intestinal fermentation pattern, cecal microbial composition, and colonic biomarkers of colorectal cancer. To fill in this gap in our knowledge, Nielsen et al. [9] fed male Sprague-Dawley rats (the initial age of 7 weeks) a basal diet (18.3% corn starch, 10% sugar, 5% cellulose, 0.3% cysteine, 3.5% mineral mix, 1% vitamin mix, 0.25% choline bitartrate, 0.014% t-butylhydroquinone, and 51.7% cooked and dried beef; as-fed basis) supplemented with 10% corn starch (HPM, control group), high-amylose potato starch (HAPS), high-amylose maize starch (HAMS), or butyrylated HAMS (HAMSB). On a dry matter basis, these four diets contained approximately 32% crude protein (~78% higher than that for adult rats [10]), 19% fat, and 4% minerals. This feeding trial, including the collection of feces, cecal digesta, and colonic tissue, lasted for 4 weeks. The results revealed that (1) the microbiota in the cecal digesta contained similar bacterial taxa among all the four dietary groups but were enriched in members of the *Ruminococcus*, *Oscillospira*, *Lactobacillus*, and *Bacteroides* genera; (2) all three RS treatments altered the cecal microbial composition in a diet-specific manner; (3) the HAMS and HAMSB diets shifted the fermentation pattern in the large intestine from protein to carbohydrate metabolism, compared with the control diet; (4) the HAMSB diet increased the concentrations (14- to 21-fold) and total amounts (20- to 53-fold) of butyrate in the feces, compared with the other diets; (5) the HAPS and HAMSB diets reduced miRNA levels for the oncogenic miR17-92 in the colonic tissue; and (6) no differences were found in the abundances of the O6MeG adduct (a cytotoxic molecule) in the colonic tissues among the four diet groups. These results suggest that dietary intakes of RS may modulate the composition and metabolism of the microbes in the large intestine of rats fed diets containing beef as the sole source of dietary protein. Because beef contains large amounts of functional AAs (e.g., taurine, glycine, and β-alanine) and small peptides (e.g., glutathione, carnosine, and anserine) [11], this food has anti-oxidative and anti-inflammatory effects in the gut [12]. Future studies are warranted to determine the interactions between RS and beef consumption and their effects on intestinal health in mammals (including humans, pigs, and rats). In nutritional studies involving the use of beef, which contains a relatively large amount of cysteine [12], as the sole or a major food protein source, the addition of 0.3% crystalline L-cysteine to the diets [2] is not necessary for meeting needs but, rather, may result in adverse effects on intestinal metabolism and health due to the excessive production of sulfur-containing metabolites such as hydrogen sulfide (H_2_S).

## 3. Effects of Dietary AAs on Intestinal Microbiota and Anti-Inflammatory Responses

AAs are substrates for the syntheses of not only tissue proteins and peptides but also low-molecular-weight substances with enormous physiological importance, including glutathione, creatine, nitric oxide (NO), polyamines (putrescine, spermidine, and spermine), heme, dopamine, histamine, and serotonin [13,14,15]. In biochemistry, AAs are classified as basic (e.g., Arg, ornithine, His, and Lys), acidic (e.g., Asp and Glu), or neutral (e.g., Ala, Asn, citrulline, Cys, Gln, Gly, Ile, Leu, Met, Phe, Pro, Ser, taurine, Thr, Trp, Tyr, and Val) according to their net charges at neutral pH. Basic, acidic, and neutral AAs account for 16.2%, 12.0%, and 71.8% of dietary AA intakes by freely living U.S. adults (both men and women), respectively; 15.8%, 14.5%, and 69.7% of dietary AA intakes by growing pigs fed a corn- and soybean-meal-based diet, respectively; 15.9%, 14.1%, and 70.0% of dietary AA intakes by growing chickens fed a corn- and soybean-meal-based diet, respectively; and 17.8%, 15.5%, and 66.7% of dietary AA intakes by largemouth bass fed fishmeal-based diets, respectively [12]. Thus, neutral AAs are quantitatively the major AAs in the diets for members of the animal kingdom, with values ranging from 67% to 72% depending on the species.

Dietary AAs are absorbed by the enterocytes of the small intestine via specific transmembrane transporters. SLC6A19 (B^0^AT1) is a major transporter for neutral AAs (including branched-chain AAs, glutamine, and tryptophan) in the intestine [16]. In addition to serving as precursors of proteins and other nitrogenous molecules, neutral AAs are used for the synthesis of glucose, triacylglycerols, or both, depending on physiological or nutritional states [12]. Thus, the expression of SLC6A19 (B^0^AT1) in the apical membrane of enterocytes may play an important role in glucose and lipid homeostasis in animals. Javed et al. [3] used SLC6A19-knockout and wild-type mice as well as untargeted metabolomic analysis to test this hypothesis. The authors found that, compared with the wild-type mice, the concentrations of the following AAs were reduced in the plasma of SLC6A19-knockout mice in a sex-dependent manner: Pro and Lys in both males and females; Cys, Gln, His, and Ser only in males but Trp and ornithine only in females. SLC6A19-knockout increased the concentrations of Ser, Asn, Cys, Gln, Gly, Ile, Leu, Met, Phe, Pro, Thr, Trp, Tyr, and Val in the urine due to the impaired reabsorption of neutral AAs from the renal tubules into the blood, despite the lack of differences in the renal expression of SLC7A7, SLC7A9, SLC3A1, and SLC1A1 between these two groups of mice. As a result of impaired absorption of neutral AAs by the small intestine and, subsequently, their increased entry into the large intestine, the concentrations of the following AAs were higher in the feces of SLC6A19-knockout mice than in those of wild-type mice in a sex-dependent manner: Leu, Trp, and Val in both males and females; acetylglutamine only in males but Ala, Gly, Ile, Lys, ornithine, Pro, and Tyr only in females. Furthermore, the concentrations of glucose were lower in the plasma of SLC6A19-knockout mice (both males and females) than in wild-type mice. Collectively, these findings support the notion that SLC6A19 (B^0^AT1) plays an important role in the absorption of neutral AAs and glucose homeostasis in animals and that sex hormones may affect whole-body AA metabolism.

Trp has multiple physiological and immunological roles in the intestinal mucosae and microbes [12]. Thus, many efforts have been directed over the past decade toward identifying the optimal dietary requirements of animals for this AA. Using weanling piglets, which are susceptible to intestinal dysfunction, Liang et al. [4] determined the role of Trp in the small-intestinal mucosal barrier and large-intestinal microbiota. Piglets were weaned at 24 days of age and fed for 4 weeks a corn- and soybean-meal-based diet supplemented with 0% (control), 0.1%, 0.2%, or 0.4% Trp. All diets were made isonitrogenous with the addition of the appropriate amount of L-alanine. The authors [4] reported that dietary supplementation with 0.2% and 0.4% Trp dose-dependently increased the concentrations of Trp in the serum of pigs. In addition, compared with the 0% Trp group, dietary supplementation with 0.2–0.4% Trp reduced the abundances of *Clostridium sensu stricto* and *Streptococcus* but increased the abundances of *Lactobacillus* and *Clostridium XI* in the jejunal digesta, as assessed with bacterial 16S rRNA gene-based high-throughput sequencing methods. Furthermore, dietary supplementation with 0.2–0.4% Trp enhanced (1) the concentrations of secretory immunoglobulin A, as well as mRNA levels for porcine β-defensins 2 and 3 in jejunal tissues; and (2) the mechanistic target of rapamycin (MTOR) cell signaling pathway, as well as the abundances of tight-junction proteins (zonula occludens 1 and 3, and claudin-1) in jejunal and duodenal tissues. Thus, dietary Trp can improve mucosal integrity, health, and function in mammals both directly by serving as a key regulator of gene expression and cell signaling and indirectly by modulating the composition of Trp-metabolizing bacteria in the small intestine.

## 4. Effects of Dietary Lipids on Intestinal Microbiota and Anti-Inflammatory Responses

Lipids (e.g., fats and fatty acids) are major macronutrients in the diet. Short- and long-chain fatty acids are the only minor metabolic fuels in the enterocytes of mammals such as pigs [17]. Trihexanoin (1,2,3-tricaproylglycerol, a short-chain triglyceride) may play important roles in the maintenance of intestinal epithelial structure and function. Using a pig model, Wu et al. [5] investigated the effects of dietary supplementation with 0.5% trihexanoin on growth performance, carbohydrate and fat metabolism, as well as intestinal morphology and function in weanling piglets. Piglets were weaned at 21 days of age to a corn- and soybean-meal-based diet. After a 3-day period of adaptation, pigs were fed the basal diet supplemented with either 0.5% soybean oil (control) or 0.5% trihexanoin for 21 days. The results revealed that trihexanoin supplementation (1) reduced the diarrhea rate during the 3-week period postweaning by 86%; (2) increased the concentrations of high-density-lipoprotein (36%) and total protein (2.5%) in the plasma on day 20 of the study; (3) decreased the concentrations of low-density-lipoprotein (−39%) on day 20 of the study, concentrations of cholesterol (−11%) on day 10 of the study, and glutamyl transpeptidase activity (−12% and −7% on days 10 and 20, respectively) in the plasma; (4) enhanced jejunal villus height (18% and 15% on days 10 and 20, respectively); (5) increased the mRNA levels and abundances of the proteins related to mucosal barrier function (e.g., claudin-1 and occluding), antioxidant capacity, and water transport capacity (aquaporin 3); and (6) altered the community of the intestinal microflora on day 21 of the study. Specifically, dietary supplementation with 0.5% trihexanoin reduced the abundances of *Enterobacteriaceae* in the digesta of the ileum (−76%), colon (−19%), and cecum (−51%); increased the abundances of *Enterococcus* (117%), *Clostridium* (228%), *Lactobacillus* (293%), and *Bifidobacterium* (240%) in the ileal digesta; and decreased the abundances of *Enterococcus* (−75%) and *Lactobacillus* (−39%) in the colonic digesta, as well as the abundances of *Clostridium* (−32%), *Lactobacillus* (−69%) and *Bifidobacterium* (−36%) in the cecal digesta. Collectively, these results indicate beneficial roles for dietary trihexanoin in improving the intestinal function and health of weanling piglets via modulating the expression of tight-junction proteins in the small-intestinal mucosa as well as the composition of microbes in the small and large intestines.

Polyunsaturated fatty acids are not only essential components of the membranes of both eukaryotes (e.g., animals) and prokaryotes (e.g., gut bacteria) but also signaling molecules in the metabolism of these organisms [18]. Of particular note, *Spirulina platensis* (a microalga) contains relatively large amounts of polyunsaturated fatty acids, particularly γ-linolenic acid (C18:3, n6), which has antimicrobial activity [19]. Some microbial species (e.g., *Firmicutes* and *Bacteroides*) in the intestine may regulate whole-body lipid metabolism and the development of obesity in mammals [20]. For example, an increase in the ratio of *Firmicutes*/*Bacteroidetes* is associated with a risk of obesity [20]. Many members (e.g., *Bacillus*, *Clostridium*, *Enterococcus*, *Lactobacillus*, and *Ruminococcus*) of the *Firmicutes* phylum (Gram-positive bacteria) break down polysaccharides (e.g., fiber and resistant starch) to lactate in the gut, whereas many members (e.g., *Bacteroides*, *Alistipes*, *Parabacteroides*, and *Prevotella*) of the *Bacteroides* phylum (Gram-negative bacteria) convert dietary fiber and protein to short-chain fatty acids in the intestine. Given the current worldwide obesity pandemic, nutritional means (e.g., consumption of *Spirulina platensis*) may beneficially alter the composition of gut microbes to ameliorate metabolic syndrome in humans and other mammals. To address this issue, Li et al. [6] used male Wistar rats fed a low-fat diet, a high-fat diet, or a high-fat diet supplemented with a *Spirulina platensis* extract isolated with 95% ethanol (SPL95). Long-term (8-week) oral administration of SPL95 (150 mg/kg body weight per day) to the rats fed the high-fat diet produced the following effects: (1) reducing white-fat gain; (2) decreasing mRNA and protein levels for the sterol regulatory-element-binding transcription factor-1c, 3-hydroxy-3-methyl glutaryl coenzyme A reductase, and acetyl CoA carboxylase pathway, but increasing those for adenosine 5′-monophosphate-activated protein kinase-α in the liver; (3) increasing the abundance of beneficial bacteria in the large intestine, including *Prevotella*, *Alloprevotella*, *Barnesiella*, *Porphyromonadaceae*, and *Paraprevotella*; and (4) decreasing the abundances of bacteria that are positively linked with dyslipidemia, such as *Turicibacter*, *Romboutsia*, *Phascolarctobacterium*, *Olsenella*, and *Clostridium XVIII*. These results indicate that SPL95 may be a functional food to provide polyunsaturated fatty acids and regulate the gut microbiota in obese individuals.

## 5. Effects of Non-Nutrient Bioactive Molecules in Diets on Intestinal Microbiota and Anti-Inflammatory Responses

Foods for mammals (e.g., humans and pigs), birds (e.g., chicken and turkeys), and aquatic animals (e.g., fish and shrimp) contain not only nutrients that are essential for their survival, growth, development, and reproduction but also some non-nutrient bioactivate molecules that can regulate gene expression, metabolic pathways, and gut homeostasis [18]. Allyl isothiocyanate (AITC) is an organosulfur compound responsible for the pungent tastes of mustard, radish, horseradish, and wasabi and reduces the production of proinflammatory cytokines such as tumor necrosis factor α and interleukin-1β via lipopolysaccharides (LPS)-stimulated immunocytes [20]. Thus, AITC may help to prevent or alleviate inflammatory bowel disease (IBD), which is characterized by chronic or recurrent inflammation of the large intestine, diarrhea, rectal bleeding, abdominal pain, fatigue, and weight loss. Using a rodent model of IBD (i.e., dextran sodium sulfate (DSS)-induced colitis), in which mice received drinking water with 2.5% DSS for 7 days before a 7-day pretreatment with 0 or 10 mg AITC/kg body weight/day, Kim et al. [7] reported an anticolitic effect of AITC, as indicated by reductions in epithelial cell loss, crypt damage, and inflammation in the gut. Furthermore, the oral administration of AITC enhanced the expression of tight-junction proteins and mucin 2 (MUC2) in the colonic tissues of DSS-challenged mice. Thus, AITC may help to overcome the limitations of the current pharmacotherapies that involve the use of anti-inflammatory drugs (e.g., corticosteroids and aminosalicylates) and immune modulators (e.g., azathioprine and 6-mercaptopurine) [21,22,23].

Intestinal damage occurs under stress conditions, particularly in neonates. This problem can be prevented or ameliorated through dietary supplementation with some functional AAs, such as glutamine, glutamate, N-acetylcysteine, or glycine [24,25,26,27]. In countries and regions where these supplements are not available, alternatives with similar nutritional efficacies must be identified. In this regard, Yi et al. [8] determined the effects of dietary supplementation with oleum cinnamomi (OCM) on the growth performance as well as intestinal morphology and functions in piglets. Piglets were weaned at 21 days of age to a corn- and soybean-meal-based diet. After a 3-day period of adaptation, pigs were fed the basal diet supplemented with 50 ppm cornstarch (control) or OCM for 21 days. The authors [8] found that, compared with the control group, dietary OCM supplementation increased (1) average daily feed intake (by 14%) and average daily weight gain (by 19%); (2) plasma concentrations of insulin (by 56%); (3) villus width and surface area in the duodenum (by 22–23%) and jejunum (by 19–29%); (4) DNA concentrations (by 96%) and RNA/DNA ratios (by 73%) in the ileum; (5) mRNA levels for epithelial growth factor receptor, Ras, extracellular signal-regulated kinase 1/2 (Erk1/2), B-cell lymphoma extra-large (Bcl-xL), villin, junctional adhesion molecule A (JAM-A), myxovirus resistance (MX) 1, MX2, and regenerating islet-derived protein 3 gamma (REG3G); (6) protein abundances of Ras and claudin-1 in the jejunal mucosa; (7) the abundances of the *Enterococcus* genus, *Lactobacillus* genus, and *Bifidobacterium* genus, as well as the abundances of the *Enterobacteriaceae* family and *Clostrium coccoides* in the colonic digesta; (8) AMP-activated protein kinase (AMPK) mRNA levels and caspase-3 protein abundance in the jejunal mucosa. Importantly, dietary supplementation with OCM decreased the incidence of diarrhea in weanling piglets by 38%. The major bioactive compound in OCM is cinnamaldehyde, which is a potent inhibitor of bacteria, yeast, and filamentous molds [28,29]. These results reveal that dietary OCM can regulate the composition of the intestinal microbiota, while improving intestinal integrity and function in weanling pigs. Thus, OCM is an effective feed additive and alternative to feed antibiotics for improving intestinal health in swine.

## 6. Impacts

Papers published in this Special Issue of *International Journal of Molecular Sciences* entitled “Nutrition and Gut Health” lay a framework for subsequent studies in this rapidly expanding research field. Examples include the impacts of dietary carbohydrates [30,31,32], AAs [33,34,35], lipids [36,37,38], and non-nutrient bioactive molecules [39,40,41] on the composition, metabolism, and function of the intestinal microbiota as well as the health, well-being, and immune response of humans and animals (e.g., pigs, poultry, rodents, fish, and crustaceans). In addition, these articles contributed to the emerging research for establishing the needs of aquatic animals (e.g., fish) for dietary AAs (e.g., glutamate and glycine) [42,43,44], lipids [45], and flavonoid- and phenolic-rich prebiotics [46] so as to optimize mucosal immune responses as well as anti-oxidative and anti-inflammatory capacities. This new knowledge is expected to facilitate the development of functional nutrients and foods to improve the health, growth, development, and reproduction of mammals, birds, and other animal species.
